# Against all odds: a tale of marine range expansion with maintenance of extremely high genetic diversity

**DOI:** 10.1038/s41598-020-69374-4

**Published:** 2020-07-29

**Authors:** Joana I. Robalo, Sara M. Francisco, Catarina Vendrell, Cristina S. Lima, Ana Pereira, Benedikt P. Brunner, Mamadou Dia, Leonel Gordo, Rita Castilho

**Affiliations:** 1 MARE – Marine and Environmental Sciences Centre, ISPA Instituto Universitário, Rua Jardim do Tabaco 34, 1149-041 Lisbon, Portugal; 2 MARE – Marine and Environmental Sciences Centre, Faculdade de Ciências da Universidade de Lisboa, Lisbon, Portugal; 3 Universidade do Algarve, Campus de Gambelas, Faro, Portugal; 4 Institut Mauritanien de Recherches Océanographiques et des Pêches, 22, Nouadhibou, Mauritania; 5 Centre of Marine Sciences (CCMAR), Campus de Gambelas, Faro, Portugal

**Keywords:** Evolutionary biology, Population genetics, Marine biology

## Abstract

The displacement of species from equatorial latitudes to temperate locations following the increase in sea surface temperatures is among the significant reported consequences of climate change. Shifts in the distributional ranges of species result in fish communities tropicalisation, i.e., high latitude colonisations by typically low latitude distribution species. These movements create new interactions between species and new trophic assemblages. The Senegal seabream, *Diplodus bellottii*, may be used as a model to understand the population genetics of these invasions. In the last decades, this species has undergone an outstanding range expansion from its African area of origin to the Atlantic coast of the Iberian Peninsula, where now occurs abundantly. Mitochondrial and nuclear markers revealed a striking high haplotypic nucleotide and genetic diversity values, along with significant population differentiation throughout the present-day geographical range of the Senegal seabream. These results are not consistent with the central-marginal hypothesis, nor with the expectations of a leptokurtic distribution of individuals, as *D. bellottii* seems to be able to retain exceptional levels of diversity in marginal and recently colonised areas. We discuss possible causes for hyperdiversity and lack of geographical structure and subsequent implications for fisheries.

## Introduction

Tropicalisation, the displacement of species from equatorial latitudes to temperate locations, is one of the major reported consequences of climate changes^1–3^. The increase of sea surface temperatures (SSTs) in the last decades has promoted shifts in the distributional ranges of species (e.g.,^4,5^) with individuals moving into areas best corresponding to their physiological optimum. Additionally, the ability of a species to colonise new habitats is influenced by oceanographic currents, the existence of adequate resource availability (i.e., habitat and food) and life-history patterns (e.g., number of eggs produced, age or parental care). These movements lead to the colonization of more poleward habitats by low latitude species, and create new interactions between species and new trophic assemblages. In commercial species, these shifts due to climate change can be magnified by fishing pressures, as reported for the North Sea cod (e.g.,^6^).

Poleward colonization by organisms with a typically equatorial distribution was described almost three decades ago for terrestrial organisms in association with postglacial recolonisation routes (e.g.,^7–10^). As a general rule, organisms follow a leptokurtic distribution type, in which the majority of individuals stay at or near the original area, and only a fraction disperse to longer distances. This range extension is usually done in a stepping-stone manner, implying that each settlement has fewer individuals compared with the previous one. Theoretically, this process corresponds to multiple successive genetic founder events associated with the corresponding genetic implications of the downsize in the effective population numbers: the erosion of the genetic diversity by genetic drift induces allele loss, the “southern richness and northern purity” paradigm^11^. Additionally, the “central-marginal hypothesis”^12^ posits that populations at the centre of the distribution have higher population sizes and gene flow, and peripheral (leading edge) populations, are smaller in size, have lower genetic diversity and will be more genetically differentiated^12^.

Phylogeographic studies using mitochondrial and nuclear genes revealed that species with similar environmental requirements and life-history traits often present distinct genetic and demographic historical patterns^13,14^. Among fish species of the northeast Atlantic, we can find the co-existence of: (1) panmictic populations without latitudinal variation in genetic diversity (e.g. *Lipophrys pholis*^15^); (2) significant population structure with similar levels of genetic diversity throughout the entire species range (e.g. *Taurulus bubalis*^16^); and (3) sharp decline of genetic diversity from south (west European) to northern (Scandinavian) populations (e.g. *Pomatoschistus microps*^17^; *Symphodus melops*^18^; *Labrus bergylta*^19^). It is less common the observation of geographical range expansion of marine species without loss of genetic diversity but, for examples, see^16,20,21^. Contemporary and increasingly visible tropicalization will contribute to more records of fast range expansion events.

**Figure 1:**
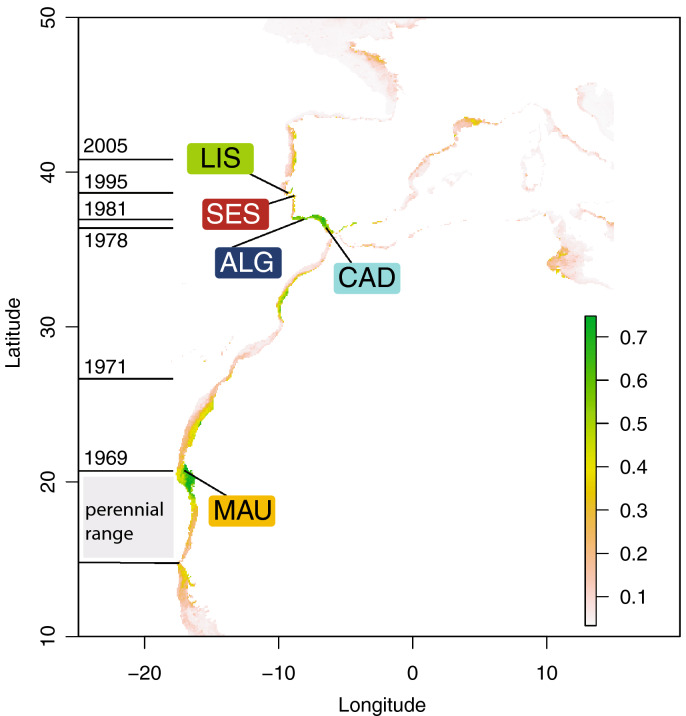
Present-day distributional range, sample locations and consensus modeled distribution of the Senegal seabream (*Diplodus bellottii*) in Northern West Africa and Iberia. Locations: MAU—Mauritania; CAD—Cadiz; ALG—Algarve; SES—Sesimbra; LIS—Lisbon. Perennial range in grey and year of first record of expanded range in horizontal lines. Color-coded sampled locations over-layed on the consensus current modeled probability of occurrence distribution (see “Material and methods” section). *Note*: The original estimated suitability value was divided by 1000 to convert the suitability value into a probability of occurrence. As a rule of thumb, sites with suitability higher than 0.5 predict presence, while sites with suitability lower than 0.5 indicate absence. Figure generated using the Biomod2 package (https://cran.r-project.org/web/packages/biomod2) implemented in the R^32^ (version 3.5.3), and Adobe Illustrator CC2019 (version 23.0.1) (https://www.adobe.com/products/illustrator.html).

The Senegal seabream, *Diplodus bellottii* Steindachner, 1882 (Sparidae), displayed an outstanding range expansion from its endemic origin (Senegal to Cape Blanco in Mauritania^22^) to the Atlantic coast of the Iberian Peninsula within a decade (see Fig. 1 for specific dates). There are no records from the archipelagos of Madeira, Canaries or Cape Verde^23^. However, its presence is reported in Mauritania^24^ and Morocco^25^ during the late 60’s. In the 1970’s, the species was consistently recorded in the Iberian Peninsula, first in Cadiz^26^ then in Algarve^27^, moving progressively towards North to Sado and Tagus (1995) estuaries^28^, and Ria de Aveiro in the north Atlantic coast of Portugal^29^ (Fig. 1). These movements are thought to result from the sea temperature rise^30^. The Tagus estuary experienced a major increase in the abundance of the Senegal seabream between 1979–1981 and 1995–1997^28^. Presently, the species is a common bycatch species of the artisanal fisheries around the Lisbon area^31^. The species is therefore a good candidate to illustrate the effect of tropicalisation on the genetic make-up of the species, and on the relationship between colonisation success and genetic diversity, without the contamination of past recolonisation events.

*Diplodus bellottii* breeding is synchronous and occurs mostly from April to June and sexual maturity is reached at 1 year old for females and two for males^31^. The same study analysed the otoliths of this species and found that the oldest specimens were 9 years old, corresponding to females with at least seven reproductive seasons^31^.

In this study, we used the documented expansion of *D. bellottii* as an opportunity to assess putative founder effects by looking for evidence of reductions in the number of mitochondrial or nuclear singleton haplotypes (haplotypes seen only once in a sample, i.e. an unshared haplotype) coherent with a predictable loss of overall genetic diversity. We hypothesise that similarly to several other marine fish species in this area of the Northeastern Atlantic such as *Halobatrachus didactylus*^33^, *Symphodus melops*^34^ or *Labrus bergylta*^19^, the recent observed range expansion will lead to a substantial loss of diversity. Also, the expanding low-density leading edge is by definition constituted by edge patches, which are most typically affected by founder events^35^, where demographic and allelic composition stochasticity^36^ is predictably stronger. If no significant reduction in singleton haplotypes is observed, and no differences among haplotype frequencies are found, there would be no evidence for a founder event or genetic bottleneck and the species may be panmictic. Alternatively, if no significant reduction in singleton haplotypes is observed but there are differences among haplotype frequencies, the species may be genetically structured. This study is the first appraisal on the genetic variability of this species across its range, a key information to understand its astonishing northward expansion. Additionally, we used species distribution modelling with present-day conditions to evaluate suitable areas for potential colonization, and explored whether the sampling coverage allowed an effective representation of the genetic diversity of the species.

## Results

A total of 357 base pairs of the mitochondrial control region were sequenced for 124 individuals, defining 118 haplotypes and 548 base pairs of the S7 first intron were resolved for 96 individuals, defining 69 haplotypes, from five sampling locations (Fig. 1). Haplotype and nucleotide diversities were high in all locations (Table 1), except in Cadiz where diversities were low. Haplotype diversities were close to 1.000 (h_CR_ = 0.997 to 1.000 and h_S7_ = 0.960 to 1.000) and nucleotide diversities were high, ranging from πCR = 0.4% (Cadiz) to 10.4% (Mauritania) and πS7 = 0.0% (Cadiz) to 2.7% (Mauritania) with πCR = 7.9 and πS7 = 0.1% for the entire data set (Table 1).

**Table 1:** Sample locations, sample abbreviations, collection dates, sample sizes and summary statistics for one mitochondrial fragment of mtDNA control region (CR) and first intron of the S7 ribosomal protein (S7) of *Diplodus bellottii*.

Location	Code	Lon	Lat	Mitochondrial D-Loop							S7						
				*N*	*n*_H_	*n*_P_	*n*_s_	*n*_P/N_	*h*	*π* (%)	*N*	*n*_H_	*n*_P_	*n*_s_	*n*_P/N_	*h*	*π* (%)
Lisbon, Portugal	LIS	38.645	− 9.233	55	50	47	43	0.94	0.997	7.77	56	28	25	19	0.89	0.960	0.08
Sesimbra, Portugal	SES	38.438	− 9.106	29	28	25	24	0.89	0.998	7.27	22	19	14	13	0.74	0.987	0.28
Algarve, Portugal	ALG	37.132	− 8.541	11	11	10	10	0.91	1.000	5.17	11	11	10	10	0.91	1.000	1.77
Cadiz, Spain	CAD	36.527	− 6.289	16	16	13	13	0.81	1.000	0.41	2	1	1	0	NA	0.000	0.00
Cape Blanc, Mauritania	MAU	20.985	− 17.003	13	13	12	12	0.92	1.000	10.37	5	5	3	3	0.60	1.000	2.74
**Total**				124	112	107	102	0.96	0.998	7.89	96	61	54	45	0.89	0.984	0.11

*Code* map and figure location codes, *Lon* longitude, *Lat* latitude, *N* number of individuals, *nh* number of haplotypes, *np* number of private haplotypes, *ns* number of singletons, *np/h* the proportion of private haplotypes, *h* haplotype diversity, *π* nucleotide diversity.

In the entire data set the three estimates of fixation *G*_*ST*_, *G′*_*ST*_, *θ* and the differentiation estimate *D* reveal significant values for CR (*G*_*ST*_ = 0.068, CI = 0.053–0.089; *G′*_*ST*_ = 0.981, CI = 0.940–0.999; *θ* = 0.045, CI = 0.032–0.062; *D* = 0.979, CI = 0.936–0.999) and for S7 (*G*_*ST*_ = 0.233, CI = 0.203–0.292; *G′*_*ST*_ = 0.990, CI = 0.979–0.998; *θ* = 0.076, CI = 0.054–0.105; *D* = 0.988, CI = 0.972–0.997). The same parameters behaved differently in pairwise comparisons for both markers (Table 2), with *G*_*ST*_ and *θ* revealing largely non-significant comparisons, and *G′*_*ST*_ and *D* showing all comparisons statistically significant. This means that haplotypes are usually distinct among the five sampling sites, with complete haplotypic differentiation (*D* = 1) involving four pairs of sampling sites in CR and six in S7, including locations that are not the most distant ones, such as Algarve and Cadiz.

**Table 2:** Pairwise CR and S7 differentiation in *Diplodus bellottii*.

Pairwise	*Gst*	95% CI	*G*′_*ST*_	95% CI	*θ*	95% CI	*D*	95% CI
**Control region**								
ALG–CAD	**0.020**	0.0027–0.0481	**1.000**	1.0000–1.0000	0.000	− 0.0324–0.0502	**1.000**	1.0000–1.0000
ALG–MAU	**0.022**	0.0011–0.0542	**1.000**	1.0000–1.0000	0.000	− 0.0394–0.0575	**1.000**	1.0000–1.0000
ALG–SES	0.016	− 0.0010–0.0389	**0.908**	0.7586–0.9395	− 0.002	− 0.0318–0.0379	**0.907**	0.7512–0.9395
ALG–LIS	**0.015**	0.0006–0.0380	**1.000**	1.0000–1.0000	0.002	− 0.0238–0.0396	**1.000**	1.0000–1.0000
CAD–MAU	0.016	− 0.0031–0.0401	**0.869**	0.6292–0.9152	− 0.005	− 0.0409–0.0394	**0.867**	0.6184–0.9152
CAD–SES	**0.012**	0.0002–0.0262	**0.918**	0.7872–0.9474	− 0.001	− 0.0237––0.0251	**0.917**	0.7834–0.9473
CAD–LIS	0.010	− 0.0003–0.0232	**0.848**	0.6895–0.9054	− 0.002	− 0.0203–0.0228	**0.847**	0.6841–0.9055
MAU–SES	**0.015**	0.0027–0.0368	**1.000**	1.0000–1.0000	0.001	− 0.0218–0.0386	**1.000**	1.0000–1.0000
MAU–LIS	0.012	− 0.0002–0.0316	**0.894**	0.7369–0.9330	− 0.001	− 0.0227–0.0329	**0.893**	0.7299–0.9330
SES–LIS	**0.008**	0.0034–0.0137	**0.962**	0.8838–0.9778	0.002	− 0.0063–0.0134	**0.961**	0.8821–0.9778
**S7**								
ALG–CAD	**0.304**	0.2773–0.3489	**1.000**	1.0000–1.0000	**0.233**	0.1961–0.2938	**1.000**	1.0000–1.0000
ALG–MAU	0.041	− 0.0119–0.1518	**1.000**	1.0000–1.0000	0.000	− 0.0847–0.1461	**1.000**	1.0000–1.0000
ALG–SES	**0.020**	0.0027–0.0510	**1.000**	1.0000–1.0000	0.005	− 0.0272–0.0578	**1.000**	1.0000–1.0000
ALG–LIS	**0.019**	0.0015–0.0462	**0.852**	0.6374–0.9218	0.010	− 0.0212–0.0568	**0.849**	0.6265–0.9219
CAD–MAU	**0.347**	0.2505–0.5787	**1.000**	1.0000–1.0000	**0.286**	0.1552–0.5641	**1.000**	1.0000–1.0000
CAD–SES	**0.292**	0.2809–0.3121	**1.000**	1.0000–1.0000	**0.221**	0.2057–0.2486	**1.000**	1.0000–1.0000
CAD–LIS	0.293	0.2851–0.3025	**1.000**	1.0000–1.0000	**0.225**	0.2149–0.2387	**1.000**	1.0000–1.0000
MAU–SES	0.032	− 0.0185–0.1418	**0.874**	0.6514–0.9268	− 0.004	− 0.0838–0.1324	**0.870**	0.6365–0.9269
MAU–LIS	0.034	− 0.0126–0.1443	**0.906**	0.7612–0.9501	0.011	− 0.0595–0.1397	**0.902**	0.7422–0.9502
SES–LIS	**0.012**	0.0023–0.0247	**0.658**	0.4418–0.8119	0.008	− 0.0105–0.0318	**0.654**	0.4348–0.8117

Values in bold represent significant values, in which the 95% confidence interval does not overlap with zero.

**Figure 2:**
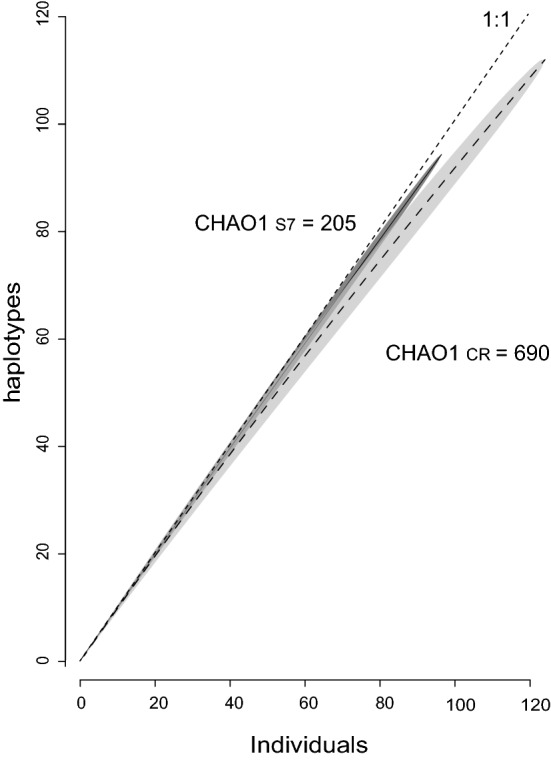
Haplotype rarefaction curves and the Chao 1 estimated total haplotypes for each marker in *Diplodus bellottii*. Shaded areas indicate 95% confidence interval from 1000 permutations. The 1:1 line indicates the maximum where each new sample represents a new haplotype. Figure generated using the chaoHaplo function (https://CRAN.R-project.org/package=spider) of the spider package implemented in R language^32^ (version 3.5.3), and Adobe Illustrator CC2019 (version 23.0.1) (https://www.adobe.com/products/illustrator.html).

The haplotype accumulation curves failed to reach the asymptote for any of the markers (Fig. 2), indicating that only part of the actual genetic diversity was captured. Both markers had largely overlapping haplotype rarefaction curves and confidence intervals, although the Chao 1 estimated total diversity for the CR region of 690 haplotypes and 206 for the S7.

**Figure 3:**
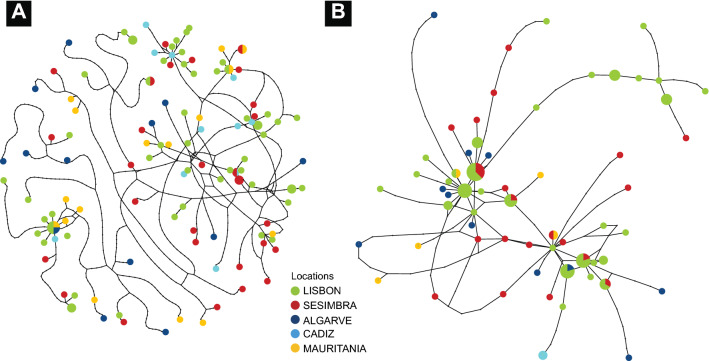
Statistical parsimony network illustrating the genealogical relationships of *Diplodus bellottii* among CR (**a**) and S7 (**b**) haplotypes (threshold of statistical significance = 95%). The size of the circle corresponds to the haplotype frequency. Pie charts indicate the proportion at which each haplotype occurs at each location. Black dots indicate the number of mutational differences separating sampled haplotypes or hypothetical (unsampled) haplotypes. Figure generated using the TCSBU^37^ and Adobe Illustrator CC2019 (version 23.0.1) (https://www.adobe.com/products/illustrator.html).

The main characteristic exhibited by the two markers’ haplotype networks is their bush-like complexity, with no central or otherwise extremely abundant haplotype. The networks displayed a large number of singleton haplotypes and very few shared haplotypes among sampling sites (coloured circles), a pattern characteristic of DNA hyperdiversity (Fig. 3). The CR region had only five shared haplotypes, with the most frequent haplotype shared among Mauritania, Cadiz and Lisbon, and the others between Lisbon–Sesimbra (the closest locations), Sesimbra–Algarve, Sesimbra–Cadiz and Lisbon–Cadiz. The S7 intron has seven shared haplotypes, four between Lisbon and Sesimbra (the closest locations), and one between Lisbon and Algarve, Lisbon and Mauritania, and Sesimbra and Mauritania.

**Figure 4:**
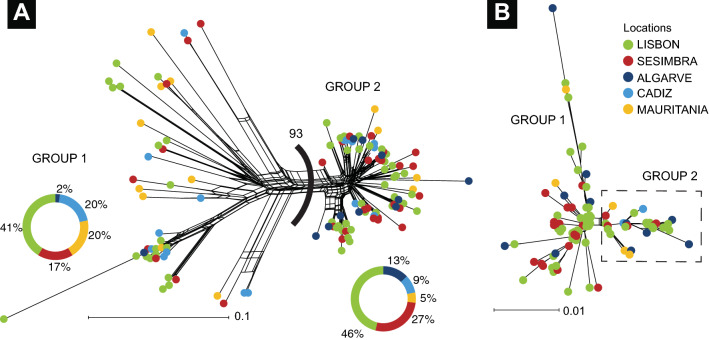
Neighbour-net networks based on 124 mitochondrial DNA CR sequences (**a**) and 96 S7 nuclear DNA (**b**) of *Diplodus bellottii*. Value between groups in CR corresponds to bootstrap replicates. Figure generated using the Splitstree^38^ and Adobe Illustrator CC2019 (version 23.0.1) (https://www.adobe.com/products/illustrator.html).

The neighbour-net network (Fig. 4) showed two lineages diverging from an unresolved polytomy, as represented by the box-like appearance of the internal branches, evident in the CR less so in the S7 network. The two lineages occurred at all sampling sites, but the χ2 test of the total frequencies of individual membership per sampling site yielded significant results only for the CR (χ2 = 10.31, *p* = 0.036) and not for the S7 (χ2 = 5.70, *p* = 0.222).

**Figure 5:**
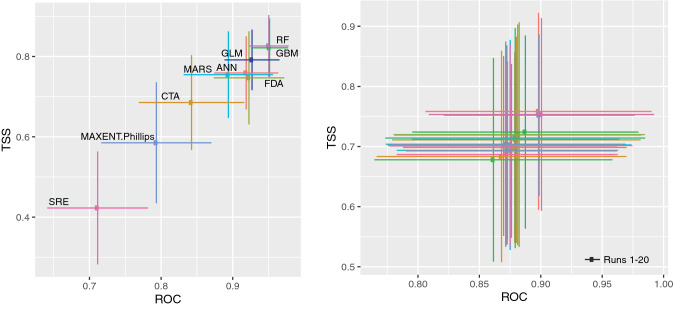
Performance statistics of all the species distribution models. Plot of the mean (dot) and standard deviation (horizontal and vertical lines) of evaluation scores for the different modeling algorithms used with Biomod2, by algorithm (**a**) or cross-validation (**b**). Best models are closer to the top-right corner of the graph. TSS: true skill statistics; ROC: area under the receiver operating characteristic curve. Figure generated using the Biomod2 package^39^ implemented in the R language^32^ (version 3.5.3), and Adobe Illustrator CC2019 (version 23.0.1) (https://www.adobe.com/products/illustrator.html).

One of the main features of the BIOMOD2 software is the ability to combine predictions made in single models in an ensemble. The species distribution modelling produced six models (ANN, FDA, GBM, GLM, MARS, and RF) that can be considered good to excellent while CTA, Maxent and SRE have a poor fit with TSS and ROC values under 0.7 (Fig. 5). Among the best performance models were RF (ROC = 0.950, TSS = 0.826) and GBM (ROC = 0.951, TSS = 0.822).

The percentage of the correctly predicted presence (sensitivity) ranged from 65 to 92% considering all models individually, with the highest value for the GBM model and the lowest for SRE (Fig. 5). Specificity (i.e., the % of absences correctly predicted) ranged between 79 and 90%, with the highest value for the MAXENT.Phillips model and the lowest for ANN. The visual assessment showed that the predictions of the consensus model reflected realistic spatial patterns of distribution of *D. bellottii* without apparent anomalies (Fig. 1). The highest probabilities of occurrence were in the Mauritania southern coast and Morocco, with discontinuous distribution patches, in contrast with south Iberia, that is associated with areas of high habitat suitability, and the Lisbon area. The overall model did not predict suitable habitats north of the Lisbon region, although the species is present in Aveiro, and predicted the area corresponding to the west Alboran Sea as a suitable habitat.

## Discussion

Remarkably high haplotype and nucleotide genetic diversities, and significant population differentiation were consistently found throughout the present-day geographical range of the Senegal seabream. These results are not consistent with the central-marginal hypothesis nor with the expectations of a leptokurtic distribution, and the Senegal seabream seems to be able to maintain very high levels of diversity in marginal and recently-colonised areas. Before dissecting these results, it is appropriate to address three main caveats regarding this work. Firstly, sample sizes are small, considering the observed hypervariability of the mitochondrial control region and even smaller in the nuclear S7, as many individuals failed to amplify. This limitation constrains the breadth of the discussion, and results are therefore interpreted with caution. Moreover, no inferences on dispersal patterns can be made from the genetic data here presented. Secondly, there is a geographic sampling gap between Mauritania and Cadiz, where the species may be present, albeit in small numbers. We could not obtain any samples from that region, and species distribution modelling may be affected by that gap. Thirdly, the small number of existing geo-referenced presence records limited the use of more than four environmental predictors to be included in the consensus prediction *D. bellottii* potential distribution model.

### Hyperdiversity in *Diplodus bellottii*

Benthopelagic fish such as *D. bellottii* usually display genetic diversity levels typically lower than pelagic species, due to differences in population sizes (e.g.^40^). Nevertheless, the diversity levels recorded in *D. bellottii* were similar to values observed in widely distributed pelagic species such as sardines (e.g.^41^) or other fish species with larger population sizes (see Table 1 in^42^). Interestingly, in the congeneric *D. vulgaris*^43^ and *D. puntazzo*^13^, the overall CR haplotype (0.983 and 0.998 respectively), and nucleotide diversity (1.8% and 4.1%) are also very high. Contrariwise, in another species of *Diplodus*, *D. sargus*, diversity measures were much lower (*h* = 0.700 and *π* = 0.8%)^13^. The differences between *D. puntazzo* and *D. sargus* genetic diversity values were attributed to a suite of fortuitous occurrences such as extinction and colonisation rather than to ecological differences between the two species^13^.

The results of the four statistics (*G*_*ST*_, *G’*_*ST*_, *θ*, and *D*) were very different with *G*_*ST*_; and *θ*, showing few pairwise significant differences, and *G’*_*ST*_, and *D* returning all pairwise comparisons significant. It is important to refer that both *θ* and *G*_*ST*_ suffer from the fact that this dataset is hypervariable, therefore restricting these estimators to values close to zero and overestimating gene flow (for a review see^44^). On the other hand, both *G’*_*ST*_ and *D*, because they measure differentiation as the fraction of a deme's population that consists of private or near-private alleles^45^, will always be significantly different from zero. The hypervariable mtDNA in *D. bellottii* leads to a lack of shared haplotypes (with rare exceptions) among locations (Supplementary Table S1), but this does not necessarily reflect population genetic differentiation by fixation of alternative haplotypes as previously reported by^46^. This result may be explained by a single or a combination of small sampling sizes and very high mutation rates. Also, effective population size and demographic stability can be contributing factors, not in the recent colonised locations which may still be locally expanding in numbers, but in the putative source, the most southern location. We have shown that with the number of singletons present, to capture all the haplotypes in the species we would need to elevate the sampling to almost 700 individuals (see Fig. 2) which makes the first explanation plausible but does not rule out the other ones.

The high diversity recorded in *D. bellottii* is mainly due to a large proportion of singletons (91% and 74%, for CR and S7, respectively) and very few among-location shared haplotypes, 4% and 13%, for CR and S7, respectively. This fact makes all locations virtually significantly different from one another, with statistically significant values of *D* ranging between 0.847–1.000 and 0.849–1.000 for CR and S7, respectively. The expanding low-density leading edge is by definition constituted by edge patches, which are typically affected by founder events^35^, where demographic^36^ and allelic composition stochasticity are predictably stronger. However, alleles present in the leading edge patches, even if absent from other marginal locations are frequently shared with sites at the centre of the distribution^47^, which is not observed in our results.

The extremely high level of genetic diversity found across the entire distribution of *D. bellottii* and the paucity of shared haplotypes throughout all the sampled locations, including the native southern site, place our findings in apparent conflict with “southern richness and northern purity”^11^ and the peripheral populations (“central-marginal hypothesis”^12^) hypotheses. Non-expected high diversity was also found in the leading edge population of other species (e.g. *Taurulus bubalis*) suggesting that marine organisms with a long planktonic stage and high numbers of colonisers can travel long distances and export their diversity to the newly colonised areas^16^. In these cases, bottlenecks may not occur because populations are not close to the carrying capacity of the habitats. *Diplodus bellottii* is a prolific species at least in its southern distribution limit^48^ and it may have sufficiently large numbers of individuals to colonise in significantly large waves of individuals.

### Possible causes of hyperdiversity

The hyperdiversity displayed by both CR and S7 markers does not automatically suggest restricted gene flow and/or population genetic differentiation. The sampling size did not capture the entire genetic diversity of the species as shown by the rarefaction curve, and did not provide a reasonable number of shared haplotypes. Moreover, abnormally high mutation rates may obscure the signal of gene flow^49^. The haplotype network (Fig. 2) with many unsampled haplotypes and haplotype accumulation curves (Fig. 4) indicate an acute subsampling of haplotypes, despite sampling sizes ranging from 11 to 55 per site, in a total of 124 specimens. As the expected diversity is ca. 690 haplotypes for the CR region and ca. 206 for the S7, many more individuals would be needed to capture a representative level of the genetic diversity of the species. It is therefore plausible that with increased sample sizes, one could detect more haplotypes in more southern locations, such as Mauritania, and shared haplotypes between these locations and the more recent colonised northern sites.

High genetic diversity in CR may have several explanations. The most plausible hypotheses involve: (1) the mutation rate of the fragment, (2) the evolutionary rates hypothesis, or (3) the metabolic rate theory (e.g.^50^). The mitochondrial control region is a high-mutation region organized in hypervariable regions flanking a central conserved region and is considered the most variant non-coding gene in fishes (e.g. for groupers^51^). This mitochondrial region is a marker of choice in phylogeographic and connectivity studies (but on the use of hyperdiverse mtDNA for population differentiation see^46^). The gastropod *Melarhaphe neritoides* revealed hypervariable mitochondrial DNA markers (16S, COI and Cytb) as a consequence of high mutation rates^49^. The second hypothesis used to explain high mutation rates in tropical areas (*D. bellottii* is considered a subtropical fish species), suggests that temperature and UV tend to generally increase mutation rates in the mtDNA of vertebrates^52^. The third hypothesis, the metabolic rate theory, posits that smaller animals accumulate mutations quicker than large animals^53,54^. However, it has been shown in the cold-water blue crab *Callinectes sapidus*, that in each recruitment event, the genetic diversity could be just as high as the one found in the adult population, although with different composition^55^. This would be exactly the opposite of a ‘sweepstakes effect,’ in which a small proportion of the gene pool contributes to the recruitment of the population^56^, therefore promoting reduced genetic diversity of populations. This way, in each seasonal recruitment, a new combination of alleles may constitute the new generation, which could help to explain the high genetic diversity in this blue crab species, even in populations near the limits of the species range^55^. Indeed, a similar pattern was found in two fish species, *Lipophrys pholis* and *Atherina presbyter*, with contrasting life-history patterns in a nursery rocky coastal area in Portugal^57^. In *L. pholis* only two out of 171 haplotypes were shared between three sampling periods, and in *A. presbyter* 25 haplotypes out of 155 were shared, which reveals an increase of private alleles in the gene pool over the years, even without a significant corresponding increase in diversity indices.

### Geographic structure

Despite the results of this study in which all sampling sites are significantly different from each other, no geographical associated structure could be inferred from the networks. Lack of geographic genetic structure can be rooted in large effective population sizes, high potential for dispersal and weak physical barriers to gene flow^58,59^, all of which possible in *D. bellottii*. Regarding population sizes, some sparids present low effective size (e.g. *Pagrus auratus,*^60^), while other species present contrasting high values (as the congeneric *D. sargus*^61^). While the dispersal distance is often used as a function of the pelagic larval duration (PLD)^62,63^, there is ample controversy on the subject, with studies finding evidence for uncorrelated individuals’ PLDs and net distance travelled^64,65^. Although the exact PLD for our species is unknown, it is expected to be around 17 days, value reported for other *Diplodus* species (e.g.^66^); this value may however vary with latitude (a proxy of sea temperature). Finally, there are no apparent physical barriers between sampled locations, which may promote considerable admixture among individuals from different origins. However, the species distribution modelling evidenced stretches of coast along northwestern Africa that seem to be an unsuitable habitat for the species, which could theoretically foster some level of geographical isolation. Apparently, *D. bellottii* overreaches those soft barriers keeping its genetic diversity undisturbed.

The concept of “spatial selection” includes the idea that more available resources coupled with less competition may lead to spatial allele sorting and increased growth at the invasion front, with the corresponding increase in reproduction^67^. Spatial selection would favour dispersal, and since both dispersal and reproductive rate limit invasion (colonisation) speed, high reproductive rates promote faster range expansions. Because *D. bellottii* is under thermal stress at 30 °C^68^, a scenario of spatial selection in the most southern range is rather probable. The reduced tolerance to higher temperatures, coupled with the species preference for estuaries with large areas and high volumes but low depth nursery grounds, means that an increase in sea surface temperatures may result in habitat loss for juveniles, as the estuaries of southern Europe may get too warm. During the summer of 2006, *D. bellottii* was first detected at 40° N (north of Portugal;^69^) showing the quick continued northward expansion of this species. Although the results from the species distribution modelling do not show a particular affinity of this species for areas north to Lisbon, the continued raising of sea surface temperature will make these areas suitable for continual range expansion of *D. bellottii.* The impacts of this species in the recently colonised ecosystems, mainly in what concerns interactions with other species occupying the same ecological niche, has not been fully evaluated. The spatial overlap of *D. bellottii* with other sparid species is not consistent, across the literature, ranging from 47%^69^ to 94%^30^. These large differences may be explained by the dynamics of the estuaries, which suffer major changes in their hydrological features across the years. A noteworthy observation is that the spatial niche overlap between *D. bellottii* and *D. vulgaris* has been shown to increase with latitude^69^.

### Origin of the two groups

The origin of the two groups (Fig. 4) is debatable and somewhat challenging to explain. When an isolation scenario is plausible, the existence of sister groups is easy to envisage. However, it is difficult to propose an allopatric (or peripatric) origin for the geographically co-distributed groups. Their distribution encompasses a continuous coastline with an apparent lack of geographical hard or soft barriers. Moreover, the observed northwards expansion of the two groups makes it difficult to conclude that they have dispersed independently along the Atlantic coastline. Without isolation, different lineages in a continuously distributed coast can arise in a large panmictic population with a long history^70^. Sometimes it is hard to realize that any particular genealogy is the single outcome of an infinite number of equally possible genealogies resulting from stochastic outcome^71^.

### Future work and management implications

This species is an important commercial species in Mauritania, but its smaller length in the Lisbon region^31^, and the existence of more valuable commercial species, has not made the Senegal seabream a main fisheries target. In Lisbon, the species is mostly a bycatch of a traditional shallow artisanal fishery^31^ and for official statistics, *D. bellottii* is merely recorded as *Diplodus* sp. The fact of this species may constitute a future target fishery should prompt an adequate biological survey to assist its proper management. The unexpected pattern of very high diversity in recently-colonised areas within a climate change scenario, deserves further investigation and this species can serve as a useful model for studying the impact of a changing environment on the genetic diversity of a coastal species. Surveys need to be implemented, ecological implications evaluated, and a genomic temporal approach assessed to understand the causes for a successful colonisation without the loss of genetic diversity levels.

## Materials and methods

### Sampling

Adult specimens of *D. bellotti* were collected from five locations along its distributional range in the northeastern Atlantic (Fig. 1 and Table 1). These sites included: Mauritania, MAU; Cadiz, CAD in South Spain; Algarve, ALG in South Portugal, and Sesimbra, SES and Lisbon, LIS in Central Portugal. After asserting the species identification, fin clips were provided by fishermen. Samples were preserved in 96% ethanol.

### DNA extraction, amplification and sequencing

Total genomic DNA was extracted with the REDExtract-N-Amp Kit (Sigma-Aldrich) following the manufacturer's instructions. The mitochondrial control region (CR) and the first intron of the nuclear S7 ribosomal protein gene (S7) were amplified, with the same kit, in a Bio-Rad Mycycler thermal cycler, using L-pro1 and H-DL1^72^, and S7RPEX1F and S7RPEX2R^73^, using the following PCR conditions: initial denaturation at 94 °C for 7′, followed by 35/30 cycles (denaturation at 94 °C for 30/45ʺ, annealing at 55 °C for 30/45ʺ, and extension at 72 °C for 1′; values CR/S7, respectively) and a final extension at 72 °C for 7′. The same primers were used for the sequencing reaction, and the PCR products were purified and sequenced in STABVIDA (Sanger sequencing, https://www.stabvida.net/).

Chromatograms were edited with Codon Code Aligner (Codon Code Corporation, https://www.codoncode.com/index.htm) and sequences were aligned with Clustal X 2.1^74^. For the S7 gene forward and reverse strands were obtained and some samples were sequenced twice for error checking. All sequences were deposited in GenBank (Accession Numbers MF120288-MF120372; and MF120408-MF120464, respectively to CR and S7).

### Genetic diversity and geographic structure

The standard measures of genetic diversity were computed for the five location sites separately and after pooling (referred to as “total population”). We used a variety of packages developed for R v.3.6.1^32^ to estimate population genetic statistics. We used *pegas*^75^ R-package^32^ to estimate standard descriptive measures of genetic diversity, including number of haplotypes and private haplotypes, haplotype diversity (h)^76,77^ and nucleotide diversity (π)^76^ and respective standard deviations.

To assess population structure, we estimated *F*_*ST*_‐like statistics^78^, such as: (1) Nei's *G*_*ST*_,^77^, (2) Hedrick's *G*′_*ST*_^79^, (3) Weir and Cockerham *θ*^80^ and (4) Jost's *D*^81^ using *diveRsity* R-package^82^. While the first three estimators measure nearness to fixation, the last quantifies the relative degree of allelic differentiation^45^. We report these four estimates to provide complementary information on the genetic structure among the Senegal seabream sampled locations. The *diveRsity* R-package was used to estimate 95% confidence intervals for all estimators using 10,000 bootstrap iterations.

### Haplotype accumulation curves

The *spider* R-package^83^ was used to obtain haplotype accumulation curves by 1000 random permutation subsampling using the functions haploAccum() and plot.haploAccum() supplied in the *spider* R-package^83^ and the Chao 1 estimator^84^, with chaoHaplo() function. Haplotype accumulation curves provide a graphical way to assess the extent of haplotype sampling by representing the extent of saturation as a function of the number of individuals sampled and the number of haplotypes accumulated. Curves displaying weak asymptotic behaviour suggest further sampling is necessary to capture the genetic variation of the species, while curves with rapid saturation indicate that much of the intraspecific haplotype diversity was sampled. The Chao1 estimates the appropriate minimum sample size to account for all haplotype diversity based on the sample size and number of detected haplotypes as well as the number of singleton and doubleton sequences (those occurring once and those appearing twice) in the dataset.

### Genetic connectivity

Population genetic connectivity in *D. bellottii* was investigated by reconstructing haplotype networks using a statistical parsimony algorithm^85^, implemented in TCS v.1.21^86^. The raw output was visualized in the web implementation of tcsBU^37^. Additionally, an unrooted neighbour-net network was constructed using SplitsTree 4^87^ to investigate the genealogical relationships among haplotypes. The network was built using HKY + I + G nucleotide substitution model for both markers, as the best selected with the modelTest function of the *phangorn*^88^ R-package according to the Akaike Information Criterion^89^ and compatible with the distance algorithms available in SplitsTree 4, and equal-angle split transformation^90^ with 1000 bootstrap replicates.

### Species distribution data: occurrence data

The georeferenced locations of the observed distribution of *Diplodus bellottii* was obtained from occurrence records from two databases: OBIS (http:// www.iobis.org/) and GBIF (https://data.gbif.org/). We used 94 records of *Diplodus bellottii* compiled from the Global Biodiversity Information Facility (GBIF, https://www.gbif.org/) and 72 points lifted from the Ocean Biogeographic Information System (OBIS, http:// www.iobis.org/) and added five own records. Spurious geo-reference errors and duplications were deleted from the initial data. Presence points were limited to within 200 m depth to consider the recorded depth range for this species (between 10 and 90 m depth)^91^. Finally, the remaining 39 presence cells were georeferenced to a 0.083° × 0.083° (= 5 arc min ~ 9.2 km) grid resolution.

### Species distribution data: environmental data

The occurrence data was linked to 43 environmental variables available from Bio‐ORACLE^92^ and MARSPEC^93^ with a spatial resolution of 5 arcmin using the R package *sdmpredictors*^94^. All predictors were delimited up to 200 m depth, given the species known bathymetric preference^95^, which enhances modelling performances by limiting the extent of extrapolation.

### Species distribution data: variable selection

The first level of predictor selection, was done by reducing collinearity between predictors, with variance inflation factor (VIF)^96^ implemented in R package *uSDM*^97,98^. We used a stringent VIF value (< 2) to retain predictors, as recommended for ecological studies see^99^. The final set of variables was reduced to four (chlorophyll *a* range, cloud fraction maximum, North–South aspect and sea surface temperature range) complying with two criteria: being conceptually meaningful variables^100^ and proportion of occurrences to predictors obeying to the general rule of thumb of 10 to 1^101^ (Table 3).

**Table 3:** Environmental variables used to model current potential distribution of the Senegal seabream (*Diplodus bellottii*).

Source	Class	Designation	Description	Units	Original resolution (km)
Bio-Oracle	Climate	Chlorophyll a range	Chlorophyll A concentration indicates the concentration of photosynthetic pigment chlorophyll A	mg/m	9
Bio-Oracle	Climate	Cloud fraction maximum	Cloud fraction indicates how much of the earth is covered by clouds	%	9
MARSPEC	Climate	Sea surface temperature range	Satellite measures of sea surface temperature (SST) obtained at a 2.5 arc-minute resolution (approximately 4 km_) from Aqua-MODIS 4-micron nighttime SST Level 3 standard mapped image products, downloaded from NASA's Ocean Color website (https://oceancolor.gsfc.nasa.gov/)	Celsius	1
MARSPEC	Seascape structure	North/South Aspect	The horizontal orientation of the seafloor	Radians	1

### Species distribution data: modelling approach

We produced an ensemble approach to obtain predictions for the current distribution implemented in *biomod2* package^102^ (v. 3.3–7) for R^32^. One of the main features of the BIOMOD2 software is the ability of producing an ensemble model by combining predictions from single models nine different methods: maximum entropy (MAXENT), general linear models (GLM), general boosted models (GBM, also referred to as boosted regression trees), classification tree analysis (CTA), artificial neural networks (ANN), surface range envelope (SRE), flexible discriminant analysis (FDA), multiple adaptive regression splines (MARS), random forests (RF), with default settings^39^. Explicit *D. bellottii* absence records were not available so we used two sets of pseudo-absences (background data) extracted at random in a proportion of ten pseudo-absences per presence cell^103^. Data (presences and pseudo-absences) were split at random into a calibration (70%) and a validation set (30%). We evaluated 140 models: 2 pseudo-absence sets × 10 iterations × 7 models. Models’ performance was evaluated using two measures: the true skill statistic (TSS^104^) and the area under the receiver operating characteristic (ROC) curve (AUC)^105^. For the ensemble modeling, only those models with a TSS and ROC scores above 0.8, and 0.9, respectively (i.e., with minimum ‘good’ rating) were considered^104,106^.

All calculations and analyses were performed with R version 3.0.3, with the following R packages *raster*^107^, rgdal^108^, *dismo*^109^, *vegan*^110^, *sp*^111^, *rJava*^112^, performed in R^32^ using the R2C2 computational facility (https://rcastilho.pt/R2C2/R2C2_cluster.html).

## Supplementary information

Supplementary Table 1.

## References

[1] Vergés A (2014). The tropicalization of temperate marine ecosystems: climate-mediated changes in herbivory and community phase shifts. Proc. R. Soc. B Biol. Sci..

[2] Cheung WW, Watson R, Pauly D (2013). Signature of ocean warming in global fisheries catch. Nature.

[3] López C, Moreno S, Brito A, Clemente S (2020). Distribution of zooxanthellate zoantharians in the Canary Islands: potential indicators of ocean warming. Estuar. Coast. Shelf Sci..

[4] Perry AL, Low PJ, Ellis JR, Reynolds JD (2005). Climate change and distribution shifts in marine fishes. Science.

[5] Encarnação J, Morais P, Baptista V, Cruz J, Teodósio MA (2019). New evidence of marine fauna tropicalization off the southwestern Iberian Peninsula (southwest Europe). Diversity.

[6] Engelhard GH, Righton DA, Pinnegar JK (2014). Climate change and fishing: a century of shifting distribution in North Sea cod. Glob. Change Biol..

[7] Hewitt GM (1996). Some genetic consequencies of ice ages, and their role in divergence and speciation. Biol. J. Lin. Soc..

[8] Ibrahim KM, Nichols RA, Hewitt GM (1996). Spatial patterns of genetic variation generated by different forms of dispersal during range expansion. Heredity.

[9] Kot M, Lewis MA, van den Driessche P (1996). Dispersal data and the spread of invading organisms. Ecology.

[10] Nichols RA, Hewitt GM (1994). The genetic consequences of long distance dispersal during colonization. Heredity.

[11] Hewitt GM (2000). The genetic legacy of the Quaternary ice ages. Nature.

[12] Eckert CG, Samis KE, Lougheed SC (2008). Genetic variation across species’ geographical ranges: the central–marginal hypothesis and beyond. Mol. Ecol..

[13] Bargelloni L (2005). The Atlantic–Mediterranean transition: discordant genetic patterns in two seabream species, *Diplodus puntazzo* (Cetti) and *Diplodus sargus* (L.). Mol. Phylogenet. Evol..

[14] Bargelloni L (2003). Discord in the family Sparidae (Teleostei): divergent phylogeographical patterns across the Atlantic–Mediterranean divide. J. Evol. Biol..

[15] Francisco SM (2011). Phylogeography of the shanny *Lipophrys pholis* (Pisces: Blenniidae) in the NE Atlantic records signs of major expansion event older than the last glaciation. J. Exp. Mar. Biol. Ecol..

[16] Almada VC, Almada F, Francisco SM, Castilho R, Robalo JI (2012). Unexpected high genetic diversity at the extreme Northern geographic limit of *Taurulus bubalis* (Euphrasen, 1786). PLoS ONE.

[17] Gysels ES, Hellemans B, Pampoulie C, Volckaert FAM (2004). Phylogeography of the common goby, *Pomatoschistus microps*, with particular emphasis on the colonization of the Mediterranean and the North Sea. Mol. Ecol..

[18] Robalo JI (2012). Northern refugia and recent expansion in the North Sea: the case of the wrasse *Symphodus melops* (Linnaeus, 1758). Ecol. Evol..

[19] Almada F (2017). Historical gene flow constraints in a northeastern Atlantic fish: phylogeography of the ballan wrasse *Labrus bergylta* across its distribution range. Proc. R. Soc. Open Sci..

[20] Ramos JE (2018). Population genetic signatures of a climate change driven marine range extension. Sci. Rep..

[21] Silva G, Horne JB, Castilho R (2014). Anchovies go north and west without losing diversity: post-glacial range expansions in a small pelagic fish. J. Biogeogr..

[22] Froese, R. and Pauly, D. FishBase. World Wide Web Electron. Publ. Available from: https://www.fishbase.org/ (version 01/2020).

[23] Wirtz P (2013). The coastal fishes of the Cape Verde Islands—new records and an annotated check-list. Spixiana.

[24] Bonnet M (1969). Les sparidés des côtes nord-ouest africaines. Rev. Trav. Inst. Pêches Marit..

[25] Aloncle H (1965). Note sur un petit *Diplodus* des cotes du Maroc. Bulletin de l'Institut des Pêches Maritimes du Maroc.

[26] Rucabado J, LLoris D (1977). Sobre la presencia de Diplodus senegalensis Cadenat, 1964, en el area de afloramiento del NW de Africa (23–26 lat. N). Rapp. Exp. Cient..

[27] Monteiro C, Lam Hoai T, Lasserre G (1987). Distribution chronologique des poissons dans deux stations de la lagune Ria Formosa (Portugal). Oceanol. Acta.

[28] Cabral H, Costa M, Salgado J (2001). Does the Tagus estuary fish community reflect environmental changes?. Clim. Res..

[29] Vinagre C, França S, Cabral H (2006). Diel and semi-lunar patterns in the use of an intertidal mudflat by juveniles of Senegal sole, *Solea senegalensis*. Estuar. Coast. Shelf Sci..

[30] Horta M, Costa MJ, Cabral H (2004). Spatial and trophic niche overlap between *Diplodus bellottii* and *Diplodus vulgaris* in the Tagus estuary, Portugal. J. Mari. Biol. Assoc. U. K..

[31] Henriques, C. V. L. V. Biologia, filogeografia e história demográfica do Sargo-do-Senegal, *Diplodus bellotti* (Steindachner, 1882) no sul do Nordeste Atlântico. (2018).

[32] R Core Team. *R: A Language and Environment for Statistical Computing* (R Foundation for Statistical Computing, Vienna, Austria, 2019). https://www.R-project.org/. Accessed February 2019.

[33] Robalo JI (2013). Are local extinctions and recolonizations continuing at the colder limits of marine fish distributions? Halobatrachus didactylus (Bloch & Schneider, 1801), a possible candidate. Mar. Biol..

[34] Knutsen H (2013). Climate change and genetic structure of leading edge and rear end populations in a northwards shifting marine fish species, the corkwing wrasse (*Symphodus melops*). PLoS ONE.

[35] Excoffier L, Ray N (2008). Surfing during population expansions promotes genetic revolutions and structuration. Trends Ecol. Evol..

[36] Snyder RE (2003). How demographic stochasticity can slow biological invasions. Ecology.

[37] Múrias dos Santos A, Cabezas MP, Tavares AI, Xavier R, Branco M (2015). tcsBU: a tool to extend TCS network layout and visualization. Bioinformatics.

[38] Huson DH (1998). SplitsTree: analyzing and visualizing evolutionary data. Bioinformatics.

[39] Thuiller W, Lafourcade B, Engler R, Araújo MB (2009). BIOMOD—a platform for ensemble forecasting of species distributions. Ecography.

[40] Hauser L, Ward RD (1998). Population identification in Pelagic fish: the limits of molecular markers. Adv. Mol. Ecol..

[41] Bowen BW, Grant WS (1997). Phylogeography of the sardines (*Sardinops spp*.): assessing biogeographic models and population histories in temperate upwelling zones. Evolution.

[42] Neilson ME, Wilson RR (2005). mtDNA singletons as evidence of a post-invasion genetic bottleneck in yellowfin goby *Acanthogobius flavimanus* from San Francisco Bay, California. Mar. Ecol. Prog. Ser..

[43] Stefanni S (2015). Establishment of a coastal fish in the Azores: recent colonisation or sudden expansion of an ancient relict population?. Heredity.

[44] Meirmans PG, Hedrick PW (2011). Assessing population structure: F(ST) and related measures. Mol. Ecol. Res..

[45] Jost L (2018). Differentiation measures for conservation genetics. Evol. Appl..

[46] Fourdrilis S, Backeljau T (2019). Highly polymorphic mitochondrial DNA and deceiving haplotypic differentiation: implications for assessing population genetic differentiation and connectivity. BMC Evol. Biol..

[47] Maggs CA (2008). Evaluating signatures of glacial refugia for North Atlantic benthic marine taxa. Ecology.

[48] Sloterdijk H (2017). Composition and structure of the larval fish community related to environmental parameters in a tropical estuary impacted by climate change. Estuar. Coast. Shelf Sci..

[49] Fourdrilis S (2016). Mitochondrial DNA hyperdiversity and its potential causes in the marine periwinkle *Melarhaphe neritoides* (Mollusca: Gastropoda). PeerJ.

[50] Canales-Aguirre CB, Ferrada-Fuentes S, Galleguillos R, Oyarzun FX, Hernandez CE (2018). Population genetic structure of Patagonian toothfish (*Dissostichus eleginoides*) in the Southeast Pacific and Southwest Atlantic Ocean. PeerJ.

[51] Zhuang ZR, Yang XD, Huang XZ, Gu HX, Wei HY, He YJ, Deng L (2017). Three new piscidins from orange-spotted grouper (Epinephelus coioides): Phylogeny, expression and functional characterization. Fish Shellfish Immunol..

[52] Evans K, Gaston K (2005). Can the evolutionary-rates hypothesis explain species-energy relationships?. Funct. Ecol..

[53] Martin AP, Palumbi SR (1993). Protein evolution in different cellular environments: cytochrome b in sharks and mammals. Mol. Biol. Evol..

[54] Rand DM (1994). Thermal habit, metabolic rate and the evolution of mitochondrial DNA. Trends Ecol. Evol..

[55] Feng X, Williams EP, Place AR (2017). High genetic diversity and implications for determining population structure in the blue crab *Callinectes sapidus*. J. Shellfish Res..

[56] Hedgecock D (1994). Does variance in reproductive success limit effective population sizes of marine organisms. Genet. Evol. Aquat. Org..

[57] Francisco S, Robalo J (2015). Genetic structure and effective population size through time: a tale on two coastal marine species with contrasting life-history patterns. Phylogenet. Evol. Biol..

[58] Avise, J. C. in *Proceedings of Stock Identification Workshop* (eds Kumpf, E. H., Vaught, R. N., Grimes, C. B. & Johnson, A. G.) 105–136 (Panama City Beach, Florida, NOAA Technical Memorandum NMFS-SEFC-199).

[59] Gold JR, Richardson LR, Furman C, Sun F (1994). Mitochondrial DNA diversity and population structure in marine fish species from the Gulf of Mexico. Can. J. Fish. Aquat. Sci..

[60] Hauser L, Adcock GJ, Smith PJ, BernalRamirez JH, Carvalho GR (2002). Loss of microsatellite diversity and low effective population size in an overexploited population of New Zealand snapper (*Pagrus auratus*). Proc. Natl. Acad. Sci..

[61] Francisco SM, Robalo JI (2020). Time matters: genetic composition and evaluation of effective population size in temperate coastal fish species. PeerJ.

[62] Bremer JRA, Viñas J, Mejuto J, Ely B, Pla C (2005). Comparative phylogeography of Atlantic bluefin tuna and swordfish: the combined effects of vicariance, secondary contact, introgression, and population expansion on the regional phylogenies of two highly migratory pelagic fishes. Mol. Phylogenet. Evol..

[63] Søren F, Barber PH (2012). Theoretical limits to the correlation between pelagic larval duration and population genetic structure. Mol. Ecol..

[64] Weersing K, Toonen R (2009). Population genetics, larval dispersal, and connectivity in marine systems. Mar. Ecol. Prog. Ser..

[65] D’Aloia CC (2015). Patterns, causes, and consequences of marine larval dispersal. Proc. Natl. Acad. Sci..

[66] Raventós N, Macpherson E (2001). Planktonic larval duration and settlement marks on the otoliths of Mediterranean littoral fishes. Mar. Biol..

[67] Ochocki BM, Miller TE (2017). Rapid evolution of dispersal ability makes biological invasions faster and more variable. Nat. Commun..

[68] Vinagre C, Narciso L, Cabral HN, Costa MJ, Rosa R (2014). Thermal sensitivity of native and invasive seabreams. Mar. Ecol..

[69] Vinagre C, Cabral H, Costa M (2010). Relative importance of estuarine nurseries for species of the genus Diplodus (Sparidae) along the Portuguese coast. Estuar. Coast. Shelf Sci..

[70] Rogers AR, Harpending H (1992). Population growth makes waves in the distribution of pairwise genetic differences. Mol. Biol. Evol..

[71] Grant WS, Liu M, Gao T, Yanagimoto T (2012). Limits of Bayesian skyline plot analysis of mtDNA sequences to infer historical demographies in Pacific herring (and other species). Mol. Phylogenet. Evol..

[72] Ostellari L, Bargelloni L, Penzo E, Patarnello P, Patarnello T (1996). Optimization of single-strand conformation polymorphism and sequence analysis of the mitochondrial control region in *Pagellus bogaraveo* (Sparidae, Teleostei): rationalized tools in fish population biology. Anim. Genet..

[73] Chow S, Hazama K (1998). Universal PCR primers for S7 ribosomal protein gene introns in fish. Mol. Ecol..

[74] Larkin MA (2007). Clustal W and Clustal X version 2.0. Bioinformatics.

[75] Paradis E (2010). pegas: an R package for population genetics with an integrated–modular approach. Bioinformatics.

[76] Nei, M. in *Population Genetics & Fishery Management* (eds Ryman, N. & Utter, F. W.) 193–223 (Washington Sea Grant Program, University of Washington, 1987).

[77] Nei M (1987). Molecular Evolutionary Genetics.

[78] Wright S (1965). The interpretation of population structure by F-statistics with special regard to systems of mating. Evolution.

[79] Hedrick PW (2005). A standardized genetic differentiation measure. Evolution.

[80] Weir BS, Cockerham CC (1984). Estimating F-statistics for the analysis of population structure. Evolution.

[81] Jost L (2009). D vs. GST: response to Heller and Siegismund (2009) and Ryman and Leimar (2009). Mol. Ecol..

[82] Hastie T, Tibshirani R, Buja A (1994). Flexible discriminant analysis by optimal scoring. J. Am. Stat. Assoc..

[83] Brown SD (2012). Spider: an R package for the analysis of species identity and evolution, with particular reference to DNA barcoding. Mol. Ecol. Res..

[84] Chao A (1984). Nonparametric estimation of the number of classes in a population. Scand. J. Stat..

[85] Templeton AR, Crandall KA, Sing CF (1992). A cladistic analysis of phenotypic associations with haplotypes inferred from restriction endonuclease mapping and DNA sequence data. III. Cladogram estimation. Genetics.

[86] Clement, M., Snell, Q., Walke, P., Posada, D. & Crandall, K. TCS: estimating gene genealogies. In *HICOMB-First International Workshop on High Performance Computational Biology*, 184–190 (2002).

[87] Bryant D, Moulton V (2004). Neighbor-net: an agglomerative method for the construction of phylogenetic networks. Mol. Biol. Evol..

[88] Schliep KP (2011). phangorn: Phylogenetic analysis in R. Bioinformatics.

[89] Sakamoto Y, Ishiguro M, Kitagawa G (1986). Akaike Information Criterion Statistics.

[90] Dress AW, Huson DH (2004). Constructing splits graphs. IEEE/ACM Trans. Comput. Biol. Bioinf..

[91] Santos MN, Monteiro CC, Erzini K, Lasserre G (1998). Maturation and gill-net selectivity of two small sea breams (genus *Diplodus*) from the Algarve coast (south Portugal). Fish. Res..

[92] Assis J (2018). Bio-ORACLE v2.0: extending marine data layers for bioclimatic modelling. Glob. Ecol. Biogeogr..

[93] Sbrocco EJ, Barber PH (2013). MARSPEC: ocean climate layers for marine spatial ecology: ecological archives E094-086. Ecology.

[94] Bosch, S., Tyberghein, L., & De Clerck, O. sdmpredictors: Species distribution modelling predictor datasets. R package version 0.9. Available at: https://github.com/lifewatch/sdmpredictors (2016).

[95] Froese, R. & Pauly, D. *FishBase. World Wide Web Electronic Publication. *www.fishbase.org (version 08/2019).

[96] Neter J, Kutner MH, Nachtsheim CJ, Wasserman W (1996). Applied Linear Statistical Models.

[97] Naimi B (2015). usdm: Uncertainty analysis for species distribution models. R Package Version.

[98] Naimi B, Hamm NA, Groen TA, Skidmore AK, Toxopeus AG (2014). Where is positional uncertainty a problem for species distribution modelling?. Ecography.

[99] Steen V, Sofaer HR, Skagen SK, Ray AJ, Noon BR (2017). Projecting species’ vulnerability to climate change: which uncertainty sources matter most and extrapolate best?. Ecol. Evol..

[100] Guisan A, Zimmermann NE (2000). Predictive habitat distribution models in ecology. Ecol. Model..

[101] Harrell FE, Lee KL, Mark DB (1996). Tutorial in biostatistics multivariable prognostic models: issues in developing models, evaluating assumptions and adequacy, and measuring and reducing errors. Stat. Med..

[102] Thuiller, W., Georges, D., Engler, R., Breiner, F., Georges, M.D., Thuiller, C.W. Package ‘biomod2’. Available at: https://cran.rproject.org/web/packages/biomod2 (2016).

[103] Chefaoui RM, Assis J, Duarte CM, Serrão E (2015). Landscape metrics as indicators of coastal morphology and its use in ecological niche modelling of seagrass species. Ecol. Interface.

[104] Allouche O, Tsoar A, Kadmon R (2006). Assessing the accuracy of species distribution models: prevalence, kappa and the true skill statistic (TSS). J. Appl. Ecol..

[105] Fielding AH, Bell JF (1997). A review of methods for the assessment of prediction errors in conservation presence/absence models. Environ. Conserv..

[106] Shabani F, Kumar L, Ahmadi M (2018). Assessing accuracy methods of species distribution models: AUC, specificity, sensitivity and the true skill statistic. GJHSS Geo Geo-Sci. Eviron. Sci. Dis. Manag..

[107] Hijmans, R. J. *Package “raster”: Geographic Data Analysis and Modeling*. R package version 2.1-48. https://cran.r-project.org/web/packages/raster. (2013). Accessed February 2019.

[108] Bivand, R. *et al.**Package ‘rgdal’: Bindings for the Geospatial Data Abstraction Library*. R package version 1.4-6. https://cran.r-project.org/web/packages/rgdal. (2015). Accessed February 2019.

[109] *Package ‘dismo’: Species Distribution Modeling*. R package version 1.1-4. https://cran.r-project.org/web/packages/dismo (R package version 1.0-12) (2014). Accessed February 2019.

[110] Oksanen J (2007). The vegan package. Community Ecol. Package.

[111] Pebesma, E., Bivand, R., Pebesma, M. E., RColorBrewer, S. & Collate, A. Package ‘sp’: Classes and methods for spatial data in R. R package version 1.3-2. *R News* **5**. https://cran.r-project.org/web/packages/sp. (2012). Accessed February 2019.

[112] Urbanek, S. *Package ‘rJava’: rJava: Low-Level R to Java Interface*. R package version 0.9-11. https://cran.r-project.org/web/packages/rJava. (2019). Accessed February 2019.

